# Gameful Experience and Perceived Educational Value in a Student-Designed Medical Escape Room for Preclinical Medical Education: Cross-Sectional Study

**DOI:** 10.2196/96011

**Published:** 2026-09-21

**Authors:** Eleni Dafli, Ioannis Kountouratzis, Ioanna Doufexi, Alexandros Nikolaidis, Aggelos Kourpanidis, Odysseas Koniaris, Nikolaos Kyriakidis, Maria Nikolaidou, Sotiria Gilou, Panagiotis Antoniou, Panagiotis D Bamidis

**Affiliations:** 1 Lab of Medical Physics and Digital Innovation, School of Medicine, Aristotle University of Thessaloniki, University Campus, Thessaloniki, Central Macedonia, 54124, Greece, 30 6982107710

**Keywords:** medical education, escape rooms, gameful experience, gamification, experiential learning, serious games

## Abstract

**Background:**

Educational escape rooms are increasingly used in medical education to promote engagement, collaboration, and active problem-solving. However, existing literature varies considerably in theoretical grounding and evaluation methodology, and limited evidence exists regarding how specific dimensions of gameful experience may relate to learners’ perceived educational value.

**Objective:**

This study aimed to evaluate preclinical medical students’ perceptions of a student-designed medical escape room and to examine associations between dimensions of gameful experience and perceived educational value.

**Methods:**

A cross-sectional study was conducted during the 2023-2024 and 2024-2025 academic years among 144 preclinical medical students participating in a student-designed educational escape room. The intervention integrated foundational medical sciences, collaborative problem-solving, and simulation-based procedural tasks. Participants completed anonymous questionnaires after the activity. Perceived educational value was assessed using the Diabetes Escape Room Perception Scale (DERPS), while gameful experience was measured using the Gameful Experience Scale (GAMEX). Descriptive statistics, reliability analyses, principal component analysis with Varimax rotation, Spearman correlation analyses, one-sample Wilcoxon signed-rank tests, and multiple linear regression models were performed using IBM SPSS Statistics version 29. Statistical significance was set at an α level of .05.

**Results:**

Of the 144 participants, 140 (97.2%) completed all questionnaires. The sample was 52.1% (73/140) female, with a mean age of 20.8 (SD 1.5) years. Participants reported favorable perceptions of the escape room, with DERPS scores exceeding the neutral midpoint (mean 4.00, SD 0.50; *t*_139_=35.50; *P*<.001). The GAMEX showed high internal consistency (Cronbach α=0.89), and factor analysis supported the original 6-factor structure. Enjoyment (mean 4.75, SD 0.41), Creative Thinking (mean 4.33, SD 0.56), Activation (mean 3.99, SD 0.63), Absorption (mean 3.70, SD 0.76), and Dominance (mean 3.48, SD 0.81) were above the scale midpoint (all *P*<.001). Scores on the Absence of Negative Affect dimension (mean 2.11, SD 1.12; *P*<.001) were below the midpoint, indicating that participants reported some degree of challenge-related negative emotions during the activity. Spearman correlation analyses demonstrated positive associations between DERPS scores and Enjoyment (ρ=0.534; *P*<.001), Creative Thinking (ρ=0.467; *P*<.001), and Absorption (ρ=0.319; *P*<.001). In multivariable regression analyses, Enjoyment was associated with both overall perception scores (β=0.385; *P*<.001; *R*²=.442) and educational efficacy scores (β=0.279; *P*=.004; *R*²=.350). Dominance was related to educational efficacy scores (β=0.194; *P*=.04).

**Conclusions:**

This study extends existing research on educational escape rooms by combining a relatively large preclinical medical student sample with validated measures of learner perception and gameful experience. Unlike previous studies that primarily assessed satisfaction, the present study identified specific experiential dimensions—enjoyment and dominance—associated with perceived educational value. These findings contribute to a theory-informed understanding of game-based learning in medical education and provide practical guidance for designing immersive, learner-centered activities. These findings support the use of student-designed, digitally enriched escape rooms as engaging educational activities in preclinical medical education.

## Introduction

Game-based learning has increasingly gained traction in higher education, particularly within the health professions, as an innovative approach to support student engagement, knowledge retention, and collaborative learning [1-3]. Rooted in experiential and constructivist learning theories, game-based learning leverages immersive environments and problem-solving mechanics to promote active participation and contextualized understanding [4-6]. By simulating real-world challenges and requiring students to apply prior knowledge in a time-pressured and collaborative format, games facilitate the development of critical thinking, decision-making, and teamwork skills, competencies essential for clinical practice [7].

One of the most prominent formats within this pedagogical movement is the educational escape room. In recent years, escape rooms have emerged as engaging and innovative educational tools within health care professions, offering experiential, game-based learning environments that promote teamwork, critical thinking, and active participation. Originally designed as recreational team-based puzzle games, escape rooms have been adapted into instructional tools that merge entertainment with curriculum-based content delivery [8,9]. Educational escape rooms offer immersive, story-driven experiences in which learners work collaboratively to solve domain-specific challenges under time constraints [10]. The format has been applied to various health disciplines, including nursing, pharmacy, public health, and increasingly, medical education [11-13].

Recent evidence suggests that educational escape rooms are increasingly being adopted not only as engagement tools but also as structured pedagogical interventions designed to support clinical reasoning, teamwork, communication, and experiential learning in health care education [9,14]. Emerging studies have also highlighted the growing integration of digital technologies, simulation-based elements, and immersive storytelling within escape room designs, particularly in response to the learning preferences of contemporary medical students [15]. In parallel, recent reviews have emphasized the need for more theoretically grounded and methodologically rigorous evaluations of educational escape rooms, particularly studies employing validated psychometric instruments and larger participant samples [14].

In medical education, escape rooms are particularly promising due to their capacity to integrate cognitive and psychomotor skills. Prior studies have shown their utility in reinforcing clinical reasoning, procedural competency, and communication within both undergraduate and postgraduate contexts [16-18], but also systematically explored the adoption of validated evaluation instruments [19]. Furthermore, escape rooms promote intrinsic motivation, emotional engagement, and interprofessional collaboration, factors that may support educational outcomes and learner satisfaction [20,21]. Despite the growing interest in educational escape rooms, several important gaps remain in the literature, particularly regarding their theoretical grounding and methodological consistency. In this context, recently funded initiatives, such as the Escape4Health project [22], have contributed to the development of structured design recommendations for educational escape rooms, aiming to support more standardized and theory-informed implementations [23]. However, these recommendations also highlight the ongoing need for empirically evaluated, systematically designed interventions that extend beyond isolated or ad hoc implementations in medical education.

However, studies on educational escape rooms have generally focused on narrow disciplinary contexts, such as nursing education, or on specific topics, such as basic life support [24-26]. In addition, many interventions are limited to small-scale implementations within single academic settings. As a result, escape room designs often target specific educational levels or isolated curricular components rather than broader, integrated applications across disciplines.

Moreover, a lack of theoretical grounding and inconsistent evaluation methodologies limit the generalizability and educational rigor of existing evidence [27]. Although learner perceptions are commonly assessed in educational escape room studies, evaluation approaches remain methodologically heterogeneous, and validated multidimensional instruments are inconsistently applied. In addition, studies vary considerably in their theoretical grounding, drawing on diverse frameworks and constructs that can make findings difficult to compare. Consequently, there remains a need for more theoretically integrated investigations examining how specific dimensions of gameful experience relate to perceived educational value within medical escape room activities [28,29]. Existing escape room studies in health care education also vary considerably in their design characteristics, target populations, and educational objectives, making comparisons across studies challenging. Together with differences in evaluation approaches and theoretical framing, this heterogeneity limits the synthesis and generalizability of findings.

Thus, despite the growing interest in educational escape rooms, several gaps remain in the literature. First, many studies rely primarily on satisfaction-based or nonvalidated evaluation approaches, limiting the comparability and robustness of findings across contexts. Second, relatively few studies have systematically examined how specific dimensions of gameful experience relate to perceived educational value in medical education settings. Finally, there is limited use of multidimensional, validated instruments that allow for a more structured analysis of learner perceptions [16,21,25].

To address these gaps, the present study evaluates a student–supervisor co-designed medical escape room using validated instruments, the Diabetes Escape Room Perception Scale (DERPS) [11] and the Gameful Experience Scale (GAMEX) [27], to examine students’ perceived educational value and to explore how distinct dimensions of gameful experience contribute to these perceptions in a preclinical medical education context.

## Methods

### Study Design

This study employed a cross-sectional design to evaluate preclinical medical students’ perceptions of a student-designed educational escape room and to examine associations between gameful experience and perceived educational value. Data were collected immediately after participation in the intervention.

### Study Setting

The study was conducted in a simulation laboratory at the School of Medicine, Aristotle University of Thessaloniki, Greece. Data collection took place during the academic years 2023‐2024 and 2024‐2025, immediately following the completion of the escape room activity.

### Participants

#### Inclusion and Exclusion Criteria

Inclusion criteria consisted of enrollment in the preclinical years of the undergraduate medical curriculum and participation in the educational escape room activity. Students who did not complete the postactivity questionnaires were excluded from analyses due to incomplete data.

#### Participant Characteristics

A total of 144 preclinical medical students participated in the study, of whom 140 (97%) provided complete questionnaire data. Participants had a mean age of 20.8 (SD 1.5) years. The sample was 52% female. All participants were enrolled in the same preclinical medical curriculum and had no prior experience with medical escape room activities.

### Sampling Procedures

Participants were recruited using convenience sampling from the eligible population of preclinical medical students. Recruitment was conducted via course-related announcements delivered by faculty members. Participation was voluntary, and no incentives were provided.

No a priori power analysis was conducted. However, the achieved sample size (N=144; complete cases n=140) was considered adequate for correlational and multiple regression analyses and exceeded sample sizes commonly reported in similar educational escape room studies.

### Educational Escape Room Intervention

#### Educational Escape Room Design

The escape room was designed through a student–supervisor co-design process involving iterative cycles of development, feedback, and refinement. Medical students contributed to the design of clinical scenarios and puzzles, while faculty ensured clinical accuracy, educational alignment, and appropriate difficulty. The final intervention was implemented in a simulation laboratory setting.

The escape room followed a structured, linear narrative consisting of eight sequential educational challenges, each aligned with core preclinical medical disciplines. The scenario was based on a fictional infectious disease (“Takamuri disease”) and was designed to simulate disease progression through increasingly complex clinical and biomedical problems.

The sequence of activities was as follows: (1) measurement and interpretation of arterial blood pressure (physiology), (2) hematological interpretation of leukocyte distributions (hematology), (3) sequencing of glycolysis enzymes to unlock biochemical clues (biochemistry), (4) identification of affected organs based on clinical progression (anatomy and pathology), (5) classification and ordering of vertebrae (osteology), (6) interpretation of cellular structure disruption based on narrative clues (cell biology), (7) pH-based identification of pharmacological compounds and calculation of a treatment formula (pharmacology), and (8) a final integrated emergency scenario requiring cardiopulmonary resuscitation (CPR) on a simulation manikin combined with simultaneous team-based problem solving.

The CPR scenario represented the concluding stage of the escape room and served as a synthesis of prior tasks, integrating theoretical knowledge with basic clinical skills under time pressure. The progression between stages was strictly linear, with successful completion of each task unlocking access to the next.

Supervisors reviewed the content for clinical accuracy, educational alignment, and appropriate difficulty level, while students contributed to the design and usability aspects. The cycle was repeated until a final version of the escape room was achieved. The elective undergraduate course of “Medical Education” has acted as the ground for cultivating such pedagogical approaches and familiarizing students with the theoretical concepts. Then, short demonstrations (by video as well as actual hands-on sessions) played the role of icebreaking while a call for engagement and participation was also released to students by the academic staff. A small group of students volunteered to participate and formed the creators group. The whole design process was informed by the students-creators’ firsthand experience with the preclinical curriculum, allowing the learning objectives, level of difficulty, and game mechanics to be closely aligned with medical students’ educational needs. By adopting a peer-designed approach, the escape room promoted relevance, engagement, and authenticity, while ensuring that the content remained appropriate for the target student population.

Thus, the escape room designed and used in this study targeted medical students in their preclinical years. The primary objective was to assist medical students in acquiring basic medical knowledge and practicing basic clinical skills through an interactive, game-based learning experience. The educational content of the escape room was structured around fundamental preclinical subjects, including histology, biochemistry, hematology, anatomy, neurophysiology, and pharmacology, while each phase of the game was designed to test essential theoretical concepts relevant to these fields. Participants progressed through multiple riddles, each containing colored locked boxes that the players were required to successfully solve in order to advance. In the final room, participants were additionally asked to perform CPR on a simulation manikin as part of a game scenario in which the game master experienced a simulated cardiac arrest. This final task aimed to connect the practical clinical skills of the players with their theoretical knowledge, resulting in a meaningful and comprehensive simulated experience.

#### Learning Objectives

The educational escape room aimed to achieve the following learning objectives:

1. Knowledge acquisition: enable preclinical medical students to reinforce and apply foundational medical knowledge from key subjects, including histology, biochemistry, hematology, anatomy, neurophysiology, and pharmacology.
2. Clinical skills development: provide students with the opportunity to practice essential basic clinical skills within a realistic simulated scenario.
3. Integration of theory and practice: promote the integration of theoretical knowledge with practical clinical decision-making through problem-solving tasks and simulation-based challenges.
4. Teamwork and collaboration: foster effective teamwork by assigning interdependent roles and requiring cooperation among participants to successfully solve puzzles and complete the final challenge.
5. Communication skills: enhance verbal communication and coordination under time pressure.
6. Critical thinking and problem-solving: develop students’ analytical thinking by engaging them in sequential riddles that require logical reasoning and medical knowledge to progress.

The stated learning objectives reflect the intended pedagogical design of the escape room intervention and are included to describe its educational structure; they were not operationalized or directly assessed as study outcomes.

#### Game Mechanics and Narrative

The escape room employed a cooperative, time-limited game structure in which teams of five medical students solved a sequence of interconnected puzzles to progress through the scenario. Each puzzle required application of preclinical medical knowledge and clinical reasoning, with successful completion unlocking subsequent tasks. Progression was linear, ensuring that teams engaged with all educational components before advancing.

To promote collaboration and role interdependence, each participant was assigned a distinct role corresponding to a medical specialty, with access to uniquely colored keys or clues required to solve specific challenges. The final task integrated procedural and cognitive skills, requiring two participants to perform cardiopulmonary resuscitation on a simulation manikin while the remaining team members solved a concurrent puzzle. This design intentionally mirrored real-world clinical teamwork, emphasizing communication, coordination, and shared responsibility under time pressure.

A narrative framing involving a public health emergency was used to provide contextual coherence and enhance immersion; however, the narrative primarily functioned as a supportive engagement mechanism rather than as a learning objective in itself.

A standardized set of digital components was used consistently across all sessions, including QR codes, screen-based prompts, and audiovisual materials. Digital components were integrated alongside the physical environment to enhance interactivity and immersion, including the use of monitors and screens to deliver video briefings, audio files for scenario progression, and QR codes that provided access to clues and additional information. Interactive digital challenges and screen-based challenges were incorporated to support problem-solving and reinforce key concepts. Immediate feedback was provided through both physical cues (eg, unlocking mechanisms) and digital responses (eg, audiovisual prompts following correct or incorrect inputs), and teams were allowed limited guidance from a facilitator if progress stalled. These elements were implemented in a uniform manner across groups. No major technical issues or disruptions were observed during the delivery of the intervention.

### Procedure

The escape room was conducted in a simulation laboratory over 60 minutes, with teams of 5 preclinical medical students each. Prior to starting, participants received standardized instructions on game rules, safety, and the learning objectives of the activity. The session was facilitated by 1 faculty member and 1 student facilitator, who provided guidance only when teams were stalled.

Teams progressed through a series of puzzles and clinical tasks using preprepared materials including locked boxes, simulation manikins, and task-specific props. Each session concluded with a debriefing to reinforce learning outcomes and discuss teamwork strategies. Immediately after completion, participants completed the 2 validated self-report questionnaires (DERPS and GAMEX) to evaluate perceptions and gameful experience. All procedures adhered to institutional ethical guidelines, and participants provided informed consent prior to participation.

### Measures and Covariates: DERPS

DERPS is a 14-item validated instrument originally developed by Eukel et al [11] in the context of a diabetes-themed educational escape room. It assesses learner perceptions of engagement, teamwork, and perceived educational value using a 5-point Likert scale. In the present study, the DERPS was applied in a medical education escape room context with minor contextual adaptation in wording to align with the broader clinical content and scenario, without modification of the original scale structure or items. The instrument was used to capture generalizable dimensions of learner perception rather than diabetes-specific knowledge outcomes. Thus, although originally developed in a diabetes-specific context, DERPS was used in this study as a generic validated instrument for evaluating experiential and educational perception dimensions in escape room-based learning. No structural modifications were made, and the instrument was used strictly at the level of construct domains rather than content-specific knowledge.

### GAMEX

The GAMEX is a 27-item validated instrument measuring 6 dimensions of gameful experience: Enjoyment, Absorption, Creative Thinking, Activation, Absence of Negative Affect, and Dominance.

The dimension termed Absence of Negative Affect reflects the extent to which participants experience low levels of frustration, anxiety, or other negative emotions during the activity. Higher scores indicate fewer negative emotional experiences.

### Composite Evaluation Form

An additional 5-item evaluation form assessed overall satisfaction with the session, including perceived experience, duration, effort, location, and materials. A composite score was calculated as the mean of these items to provide an overall global indicator of participants’ appraisal of the educational session, reflecting a broad evaluative judgment across complementary aspects of the experience rather than a distinct latent psychological construct.

### Covariates

No additional covariates were included in the analyses.

### Statistical Analysis

#### Software and Preliminary Analyses

Data were analyzed using IBM SPSS Statistics version 29. Descriptive statistics (means, SDs, frequencies, and percentages) were computed. Normality and outliers were assessed using skewness and kurtosis.

#### Missing Data and Effect Sizes

Missing data accounted for 5.3% of the total values and were concentrated primarily in 6 variables, with variable-specific missingness ranging from 15% to 16.4%. To evaluate the missing data mechanism, the Little missing completely at random (MCAR) test was performed and indicated that the missingness pattern was consistent with the data being MCAR (*χ*²_81_=63.82; *P*=.92). Given that the MCAR assumption was supported, missing values were handled using complete-case analysis, resulting in a final analytical sample of 140 complete cases.

#### Reliability and Validity Analyses

Internal consistency of DERPS and GAMEX was assessed using Cronbach α (*α*≥0.70 considered acceptable). Factorial validity of GAMEX was evaluated via principal component analysis with Varimax rotation. The Kaiser-Meyer-Olkin measure and the Bartlett test of sphericity were used to assess sampling adequacy and factorability.

#### Correlation Analyses

The Spearman rank-order correlations were used to examine relationships between DERPS perception, educational efficacy scores, and GAMEX dimensions.

#### Regression Analyses

Multiple linear regression models were conducted to evaluate whether GAMEX dimensions were associated with DERPS perception and educational efficacy scores. Regression assumptions—including linearity, independence of errors, homoscedasticity, and multicollinearity—were assessed. Standardized beta coefficients (β), significance values (p), *R*², and squared semipartial correlations (sr²) were reported to quantify each factor’s unique contribution.

#### Comparative Analyses (Midpoint Testing)

One-sample 2-tailed *t* tests and Wilcoxon signed-rank tests were used to compare DERPS and GAMEX scores against the neutral midpoint of the scales (3=“neither agree nor disagree”). Statistical significance was set at *P*<.05.

#### Sample Size, Power, and Precision

No a priori power analysis was conducted; however, the achieved sample size (N=144; complete cases n=140) was considered adequate for the planned correlational and regression analyses and exceeded sample sizes commonly reported in comparable educational escape room studies.

### Ethical Considerations

The study was approved by the Institutional Review Board of Aristotle University of Thessaloniki (approval number: 3310651/2022) and conducted in accordance with the Declaration of Helsinki. All participants provided written informed consent, including permission for anonymized research use and Institutional Review Board–approved secondary analysis. Participation was voluntary with no compensation. All data were collected anonymously, deidentified prior to analysis, and reported in aggregate form. No identifiable images or personal data are included.

## Results

### Participants

**Figure 1.**
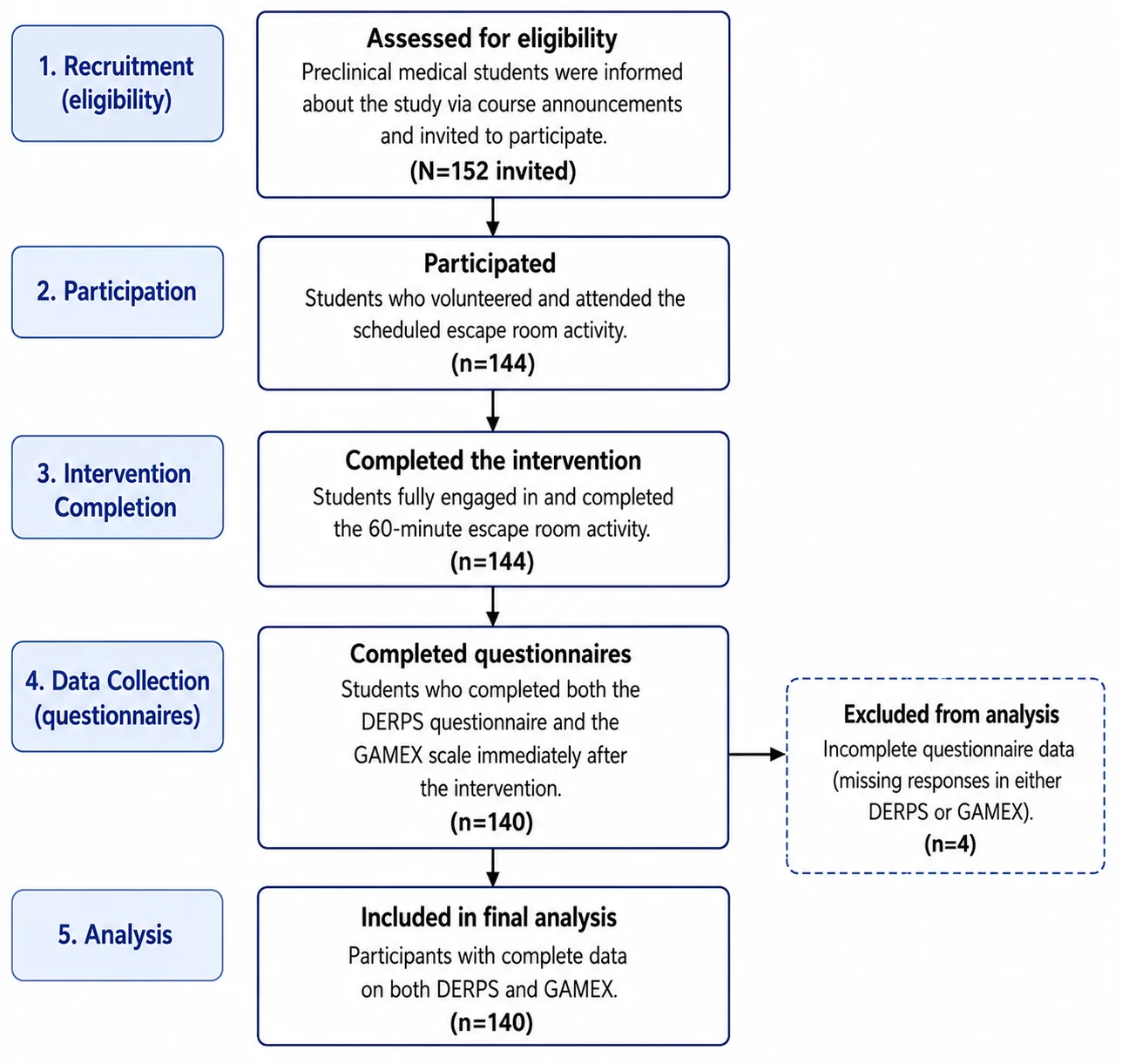
Participant flow diagram illustrating recruitment, eligibility assessment, participation, and inclusion in the final analysis of a cross-sectional study among preclinical medical students participating in a student-designed medical escape room at Aristotle University of Thessaloniki, Greece. Data were collected during the 2023‐2024 and 2024‐2025 academic years. Of 144 enrolled students, 140 completed all study questionnaires and were included in the final analysis. DERPS: Diabetes Escape Room Perception Scale; GAMEX: Gameful Experience Scale.

A total of 144 preclinical medical students participated, of whom 140 (97%) completed all study questionnaires and were included in the final analysis (Figure 1). Participants had a mean age of 20.8 (SD 1.5) years, and 52% were female. All participants were enrolled in the same preclinical medical curriculum, and none reported prior experience with medical escape rooms.

### DERPS Perception Scores

Participants reported significantly positive perceptions of the escape room activity. The overall mean DERPS score was 4.00 (SD 0.50), with a mean difference from the neutral midpoint of 1.23 (95% CI 1.16-1.30; *t*_139_=35.50; *P*<.001). Internal consistency was acceptable (Cronbach *α*=0.71). Item-level responses are presented in Table 1. The highest-rated items were recommending the activity to other students (mean 4.90, SD 0.40) and general enjoyment of games (mean 4.60, SD 0.60).

**Table 1.** Item-level responses of the Diabetes Escape Room Perception Scale (DERPS) assessing perceived educational value and collaborative learning experience among 140 preclinical medical students participating in a student-designed medical escape room^a^.

Item	Mean (SD)	“Strongly Disagree” (%)	“Disagree” (%)	“Neutral” (%)	“Agree” (%)	“Strongly Agree” (%)
The escape room encouraged me to think about material in a new way	4.20 (0.80)	1.40	0.7	13.6	45.7	38.6
I would recommend this activity to other students	4.90 (0.40)	0	0	0.7	13.6	85.7
I learned from my peers during the escape room	4.3 (0.80)	0.7	2.1	10.7	37.1	49.3
The escape room was an effective way to review the topic of leadership	4.1 (0.80)	0.7	4.3	15.8	46.8	32.4
The escape room was an effective way to learn new information related to leadership	4 (0.80)	0	2.9	22.9	45.7	28.6
I learn better in a game format than in a didactic lecture	4.4 (0.80)	0.7	1.4	14.3	28.6	55
The escape room was an effective way to assist my learning of the criteria I apply for prioritizing in challenging situations	4.4 (0.70)	0	0.7	7.9	40.3	51.1
The escape room was an effective way to assist my learning of managing different communication channels when sending and receiving information	4.3 (0.60)	0	0.7	7.9	50	41.4
The escape room was an effective way to assist my learning of dealing with emotional challenges arising from conflicts	3.9 (1.00)	1.4	5	28.8	34.5	30.2
I feel I was able to engage with my teammates to learn new material	4.3 (0.90)	1.7	1.7	11.8	38.7	46.2
It was difficult for me to focus on learning because I was feeling stressed or overwhelmed	3.8 (1.30)	9.2	6.7	16	31.9	36.
The noneducational portions (eg, cyphers, puzzles, etc) did not distract me from learning about leadership	3.9 (1.30)	9.3	7.6	7.6	34.7	40.7
I prefer assembling information from a variety of sources when learning new material	4.2 (0.80)	0.9	1.7	12.8	45.3	39.3
In general, I enjoy playing games (video games, board games, social media games, etc)	4.6 (0.60)	0	0	7.6	28	64.4

a Cross-sectional study conducted at Aristotle University of Thessaloniki, Greece, during the 2023‐2024 and 2024‐2025 academic years.

### Postsession Evaluation and Association With DERPS Scores

It should be noted that the composite evaluation score, DERPS, and GAMEX dimensions all rely on self-report and share conceptual overlap around subjective experience and satisfaction. The associations reported in this and subsequent sections should therefore be interpreted as descriptive and exploratory rather than indicative of independent causal effects. Shared method variance may contribute to the observed effect sizes.

Participants reported high overall satisfaction across all five evaluation items (Table 2). A statistically significant moderate positive association was observed between the composite evaluation score and DERPS perception scores (ρ=0.421, 95% CI 0.268-0.560; *P*<.001), and this association was consistent across individual evaluation items (Table 3). In a simple linear regression, the composite evaluation score explained 25.1% of the variance in DERPS scores (*R*²=.251), indicating that more favorable session appraisals were associated with higher perceived educational value.

**Table 2.** Item-level descriptive statistics of the postactivity evaluation form assessing overall experience, satisfaction, effort, and learning environment adequacy among 140 preclinical medical students following participation in a student-designed medical escape room^a^.

Item	Mean (SD)	“Strongly Disagree” (%)	“Disagree” (%)	“Neutral” (%)	“Agree” (%)	“Strongly Agree” (%)
I was satisfied with today’s experience.	4.80 (0.50)	0	0	2.2	15.3	82.5
The duration of the session was appropriate.	4.70 (0.50)	0	0	3.6	23.2	73.2
The effort required during the session was appropriate.	4.50(0.70)	0	0	10.1	25.4	64.5
Τhe location where the session took place was adequate.	4.20 (1.00)	1.4	5.8	13	32.6	47.1
The materials used in the session were appropriate.	4.60 (0.70)	0.7	0.7	2.9	31.2	64.5

a Cross-sectional study in Greece (Aristotle University of Thessaloniki), during the 2023‐2024 and 2024‐2025 academic years.

**Table 3.** Spearman rank-order correlations between individual postactivity evaluation items and Diabetes Escape Room Perception Scale (DERPS) scores among 140 preclinical medical students after participation in a student-designed medical escape room intervention in Greece (Aristotle University of Thessaloniki, 2023‐2025)^a^.

Individual evaluation item	Spearman ρ^b^ (95% CI)	*P* value
Composite evaluation score	0.421 (0.268-0.560)	<.001
I was satisfied with today’s experience.	0.417 (0.255-0.559)	<.001
The duration of the session was appropriate.	0.221 (0.056-0.375)	.01
The effort required during the session was appropriate.	0.347 (0.173-0.487)	<.001
Τhe location where the session took place was adequate.	0.303 (0.143-0.451)	<.001
The materials used in the session were appropriate.	0.347 (0.198-0.487)	<.001

a Statistical significance was set at *P*<.05.

b Spearman rank-order correlation coefficients are reported.

### Gameful Experience (GAMEX) — Descriptive Results

The GAMEX scale demonstrated high internal consistency (Cronbach *α*=0.89). Subscale reliability values were comparable to those reported in the original validation study (Table 4). Enjoyment (mean 4.75, SD 0.41) and Creative Thinking (mean 4.33, SD 0.56) were the highest-rated dimensions, followed by Activation (mean 3.99, SD 0.63), Absorption (mean 3.70, SD 0.76), and Dominance (mean 3.48, SD 0.81). Absence of Negative Affect was the lowest-rated dimension (mean 2.11, SD 1.12), indicating that participants experienced some degree of challenge-related negative emotions during the activity (Table 5).

**Table 4.** Internal consistency reliability (Cronbach α) of the Gameful Experience Scale (GAMEX) dimensions in a cross-sectional study of 140 preclinical medical students participating in a student-designed medical escape room at Aristotle University of Thessaloniki, Greece (2023‐2024 and 2024‐2025), compared with values reported in the original validation study of the GAMEX scale (Eppmann et al [27]).

Dimension	α (this study)	α (original GAMEX validation study)
Enjoyment	0.91	0.96
Absorption	0.85	0.91
Creative Thinking	0.84	0.88
Activation	0.70	0.87
Absence of Negative Affect	0.83	0.85
Dominance	0.79	0.84
Total	0.89	0.89

**Table 5.** Descriptive statistics (mean, SD) for Gameful Experience Scale (GAMEX) dimensions measuring Enjoyment, Absorption, Creative Thinking, Activation, Absence of Negative Affect, and Dominance among 140 preclinical medical students participating in a student-designed medical escape room intervention in Greece, during the 2023‐2024 and 2024‐2025 academic years.

Items	Mean (SD)
GAMEX total	
GAMEX-enjoyment	4.75 (0.41)
GAMEX-absorption	3.70 (0.76)
GAMEX-creative thinking	4.33 (0.56)
GAMEX-activation	3.99 (0.63)
GAMEX-absence of negative affect	2.11 (1.12)
GAMEX-dominance	3.48 (0.81)

One-sample Wilcoxon signed-rank tests confirmed that Enjoyment, Absorption, Creative Thinking, Activation, and Dominance were all significantly above the neutral midpoint (all *P*<.001), while Absence of Negative Affect was significantly below it (*P*<.001), indicating minimal but present negative emotional experience (Table 6).

**Table 6.** One-sample Wilcoxon signed-rank tests comparing Gameful Experience Scale (GAMEX) dimension scores against the neutral midpoint (3) in a cross-sectional sample of 140 preclinical medical students participating in a student-designed medical escape room in Greece during the 2023‐2024 and 2024‐2025 academic years.

GAMEX dimension	Median (IQR)	*z* score	*P* value	Direction
Enjoyment	5 (4.67-5.0)	–10.503	<.001	Median >3
Absorption	3.5 (3.17-4.33)	–8.127	<.001	Median >3
Creative Thinking	4.25 (4.0-5.0)	–10.248	<.001	Median >3
Activation	3.75 (3.5-4.38)	–9.937	<.001	Median >3
Absence of Negative Affect	2.0 (1.33-2.67)	–6.943	<.001	Median <3
Dominance	3.5 (3.0-4.0)	–5.464	<.001	Median >3

Intercorrelations among GAMEX dimensions are presented in Table 7. Strong positive associations were observed between Creative Thinking and Absorption (ρ=0.542; *P*<.001) and between Creative Thinking and Activation (ρ=0.606; *P*<.001), while Absence of Negative Affect was weakly and negatively associated with Enjoyment (ρ=−0.216; *P*=.01).

**Table 7.** Spearman correlation matrix between Gameful Experience Scale (GAMEX) dimensions assessing gameful experience in a cross-sectional study of 140 preclinical medical students following participation in a student-designed escape room in Greece (Aristotle University of Thessaloniki, during the 2023‐2024 and 2024‐2025 academic year).

Dimension	Enjoyment	Absorption	Creative Thinking	Activation	Absence of Negative Affect	Dominance
Enjoyment						
Spearman ρ	—^a^					
*P* value	—					
Absorption						
Spearman ρ	0.316^b^	—				
*P* value	<.001	—				
Creative Thinking						
Spearman ρ	0.439^b^	0.542^b^	—			
*P* value	<.001	<.001	—			
Activation						
Spearman ρ	0.142	0.551^b^	0.606^b^	—		
*P* value	.09	<.001	<.001	—		
Αbsence of Negative Affect						
Spearman ρ	–0.216^c^	0.086	–0.009	0.229^d^	—	
*P* value	.01	.31	.91	.007	—	
Dominance						
Spearman ρ	0.133	0.273^d^	0.265^d^	0.239^d^	0.314^b^	—
*P* value	.15	.003	.004	.01	<.001	—

a Not applicable.

b *P*<.001.

c *P*<.05.

d *P*<.01.

### Associations Between GAMEX Dimensions and Overall Perception Scores (Primary Outcome)

Spearman correlation analyses revealed significant positive associations between DERPS overall perception scores and Enjoyment (ρ=0.534, 95% CI 0.393-0.651; *P*<.001), Creative Thinking (ρ=0.467, 95% CI 0.330-0.587; *P*<.001), Absorption (ρ=0.319, 95% CI 0.151-0.469; *P*<.001), and Activation (ρ=0.183, 95% CI 0.018-0.338; *P*=.03). A weak negative association was observed for Absence of Negative Affect (ρ= −0.170, 95% CI −0.338 to 0.005; *P*=.045). Dominance was not significantly associated with overall perception scores (ρ=0.146, 95% CI −0.052 to 0.332; *P*=.12). Full correlation results are presented in Table 8.

**Table 8.** Spearman correlations between Gameful Experience Scale (GAMEX) dimensions and Diabetes Escape Room Perception Scale (DERPS) scores assessing perceived educational value of a student-designed medical escape room among 140 preclinical medical students in Greece (cross-sectional study, during the 2023‐2024 and 2024‐2025 academic years).

GAMEX dimension	Spearman ρ (95% CI)	*P* value
Enjoyment	0.534^a^ (0.40 to 0.65)	<.001
Creative Thinking	0.467^a^ (0.32 to 0.60)	<.001
Absorption	0.319^a^ (0.14 to 0.48)	<.001
Activation	0.183^b^ (0.02 to 0.34)	.03
Negative Affect	–0.170^b^ (−0.33 to 0.00)	.045
Dominance	0.146 (−0.05 to 0.34)	.12

a *P*<.01.

b *P*<.05.

**Figure 2.**
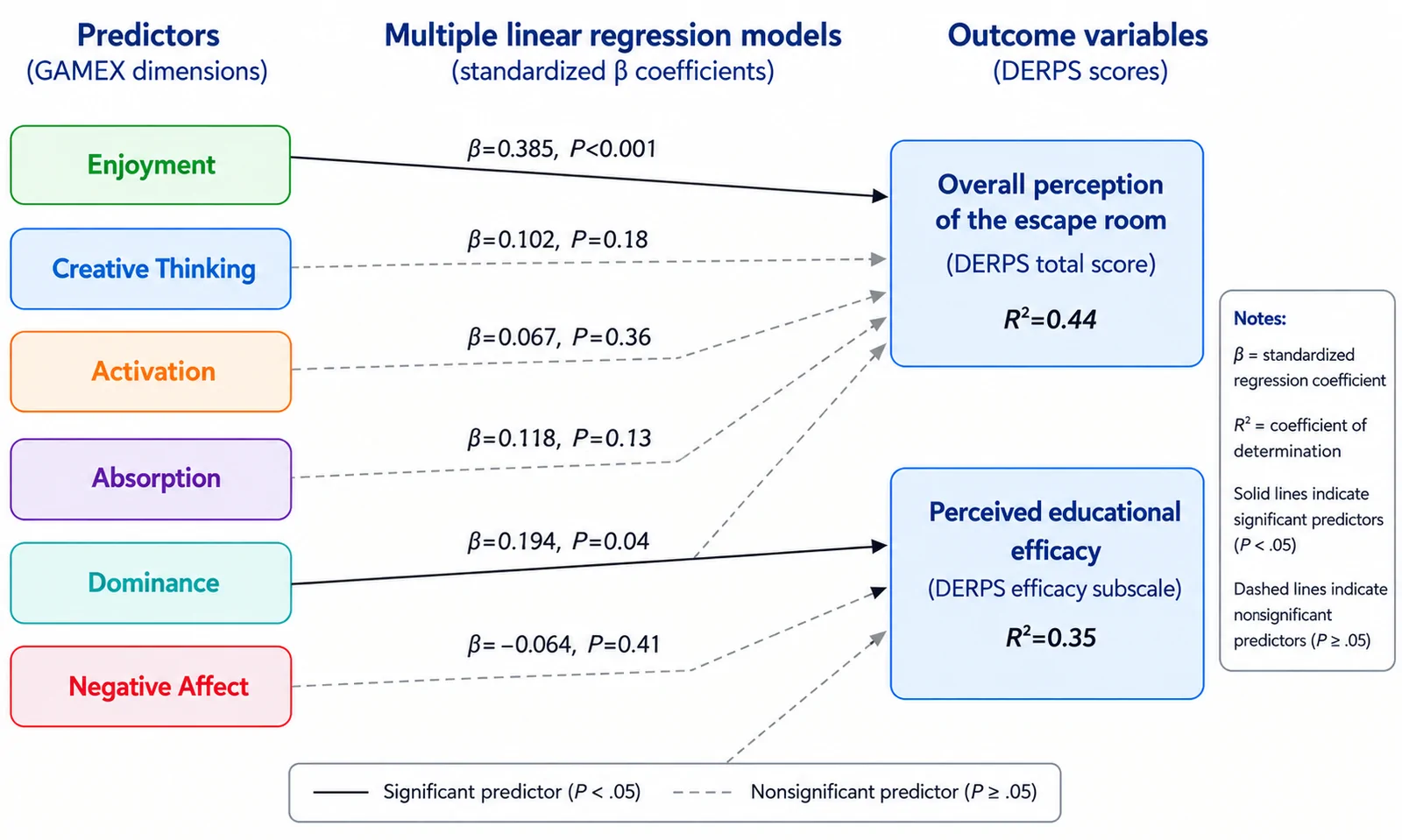
Conceptual multiple linear regression model illustrating associations between Gameful Experience Scale (GAMEX) dimensions (Enjoyment, Absorption, Creative Thinking, Activation, Absence of Negative Affect, and Dominance) and perceived educational value (Diabetes Escape Room Perception Scale [DERPS] scores) in a cross-sectional study of 140 preclinical medical students participating in a student-designed medical escape room at Aristotle University of Thessaloniki, Greece, during the 2023‐2024 and 2024‐2025 academic years.

In a multiple linear regression model, GAMEX dimensions collectively explained 44.2% of the variance in overall perception scores. Enjoyment was the strongest contributor (β=0.385, *P*<.001; sr²=10.2%), followed by Creative Thinking (β=0.269; *P*=.008; sr²=3.8%) and Absence of Negative Affect (β= −0.216; *P*=.02; sr²=2.9%). Absorption, Dominance, and Activation did not contribute significantly (*P=*.17, *P=*.24, and *P=*.51, respectively). Full regression results are presented in Table 9, and the pattern of associations is illustrated in Figure 2.

**Table 9.** Multiple linear regression analysis examining associations between Gameful Experience Scale (GAMEX) dimensions and Diabetes Escape Room Perception Scale (DERPS) scores among 140 preclinical medical students participating in a student-designed medical escape room at Aristotle University of Thessaloniki, Greece. Cross-sectional study conducted during the 2023‐2024 and 2024‐2025 academic years.

Dimensions	B (95% CI)	β coefficient	*t* test (*df*)	*P* value	Part corr	sr² (Part²), %
Enjoyment	0.395 (0.220 to 0.570)	0.385	4.47 (109)	<.001	0.320	10.2
Creative Thinking	0.203 (0.054 to 0.352)	0.269	2.71 (109)	.008	0.194	3.80
Absence of Negative Affect	−0.081 (−0.149 to −0.013)	−0.216	−2.36 (109)	.02	−0.169	2.90
Absorption	0.070 (−0.032 to 0.172)	0.131	1.37 (109)	.17	0.098	0.96
Dominance	0.051 (−0.035 to 0.136)	0.098	1.18 (109)	.24	0.084	0.71
Activation	−0.045 (−0.177 to 0.088)	−0.069	−0.67 (109)	.51	−0.048	0.23

### Associations Between GAMEX Dimensions and Educational Efficacy Scores (Secondary Outcome)

Spearman correlation analyses indicated that DERPS educational efficacy scores were significantly and positively associated with Enjoyment (ρ=0.388, 95% CI 0.227-0.530; *P*<.001), Creative Thinking (ρ=0.447, 95% CI 0.294-0.584; *P*<.001), Absorption (ρ=0.389, 95% CI 0.221-0.539; *P*<.001), Activation (ρ=0.307, 95% CI 0.147-0.459; *P*<.001), and Dominance (ρ=0.361, 95% CI 0.157-0.540; *P*<.001). Absence of Negative Affect was not significantly associated with educational efficacy scores (ρ=0.120, 95% CI −0.060 to 0.297; *P*=.16). Full results are presented in Table 10.

**Table 10.** Spearman correlations between Gameful Experience Scale (GAMEX) dimensions and Diabetes Escape Room Perception Scale (DERPS) educational efficacy subscale scores assessing perceived learning outcomes in a cross-sectional study of 140 preclinical medical students participating in a student-designed escape room at the Aristotle University of Thessaloniki, Greece, during the 2023‐2024 and 2024‐2025 academic years.

GAMEX dimension	Spearman ρ^a^ (95% CI^b^)	*P* value
Enjoyment	0.388^c^ (0.227 to 0.530)	<.001
Creative Thinking	0.447^c^ (0.294 to 0.584)	<.001
Absorption	0.389^c^ (0.221 to 0.539)	<.001
Activation	0.307^c^ (0.147 to 0.459)	<.001
Negative Affect	0.120 (−0.060 to 0.297)	.16
Dominance	0.361^c^ (0.157 to 0.540)	<.001

a Spearman rank-order correlation coefficient.

b CIs were estimated using 5000 bootstrap samples with bias-corrected and accelerated (BCa) adjustment.

c *P*<.01.

In a multiple linear regression model, GAMEX dimensions collectively explained 35.0% of the variance in educational efficacy scores. Enjoyment was again the strongest contributor (β=0.279; *P*=.004; sr²=5.33%), while Dominance also emerged as a significant factor (β=0.194; *P*=.04; sr²=2.66%). Creative Thinking showed a marginal but nonsignificant contribution (β=0.203; *P*=.06). The CIs for Enjoyment and Dominance excluded zero, supporting the robustness of these associations. Absorption, Activation, and Absence of Negative Affect did not contribute significantly, with CIs including zero. All predictors demonstrated acceptable multicollinearity (variance infation factor<2.1). Full regression results are presented in Table 11.

**Table 11.** Multiple linear regression analysis relating Diabetes Escape Room Perception Scale (DERPS) educational efficacy scores to Gameful Experience Scale (GAMEX) dimensions in a cross-sectional sample of 140 preclinical medical students participating in a student-designed medical escape room at Aristotle University of Thessaloniki, Greece, during the 2023‐2024 and 2024‐2025 academic years.

Dimensions	B (95% CI)	β coefficient	*t* test (*df*)	*P* value	Part corr	sr² (Part²), %
Enjoyment	0.396 (0.130 to 0.661)	0.279	2.95 (106)	.004	0.276	5.33
Absorption	0.103 (−0.052 to 0.258)	0.137	1.32 (106)	.19	0.127	1.07
Creative Thinking	0.212 (−0.012 to 0.437)	0.203	1.87 (106)	.06	0.179	2.16
Activation	−0.004 (−0.204 to 0.197)	–0.004	–0.04 (106)	.97	–0.003	0
Negative Affect	0.036 (−0.068 to 0.141)	0.070	0.69 (106)	.49	0.067	0.29
Dominance	0.138 (0.007 to 0.270)	0.194	2.09 (106)	.04	0.199	2.66

## Discussion

### Principal Findings

This study evaluated a student-supervisor co-designed medical escape room for preclinical medical students and examined how different dimensions of gameful experience relate to perceived educational value. The data were collected across 2 consecutive academic years (2023‐2024 and 2024‐2025) from different cohorts of preclinical medical students, and therefore represent a pooled cross-sectional sample rather than a longitudinal follow-up of the same participants. The dimensions of gameful experience were differentially associated with educational perceptions, with enjoyment and dominance emerging as the strongest contributors to students’ evaluations of the activity. These findings suggest that educational escape rooms can provide engaging learning experiences while highlighting specific experiential factors that may shape students’ perceptions of educational value, within the limits of self-reported, cross-sectional data, consistent with prior research [30,31].

In the present study, 2 DERPS-related but distinct outcomes were examined. The overall DERPS score reflected students’ general perceptions of the escape room experience, whereas the DERPS educational efficacy subscale specifically assessed perceived educational value. These outcomes are interpreted separately throughout the discussion. Students reported significantly positive perceptions of the escape room activity, with mean DERPS scores exceeding the neutral midpoint. This pattern was consistent across parametric and nonparametric analyses, indicating robust endorsement of the activity’s perceived educational value. These findings align with prior research demonstrating that collaborative, experiential gamified formats can foster meaningful and engaging learning experiences in health professions education and related fields [11,32,33].

Analysis of gameful experience using the GAMEX scale further indicated that the escape room successfully elicited elevated levels of positive experiential dimensions. Enjoyment, Absorption, Creative Thinking, Activation, and Dominance were all rated significantly above the neutral midpoint, suggesting that students experienced the activity as engaging, cognitively stimulating, immersive, and competence-supportive. In contrast, Absence of Negative Affect was rated significantly below the midpoint, indicating that students reported some degree of challenge-related negative emotions during the activity. This dimension reflects the extent to which participants experience low levels of frustration or anxiety, with lower scores indicating a greater presence of such emotions. This experiential profile, characterized by high positive engagement alongside some degree of emotional challenge, is consistent with theoretical models of game-based learning that recognize challenge and moderate affective arousal as inherent and potentially productive features of immersive learning environments [3-6].

A focused educational efficacy subscale of the DERPS was constructed, allowing conceptual separation between experiential enjoyment and perceived instructional value. Correlation analyses suggested that most GAMEX dimensions were positively associated with both overall perception scores and educational efficacy scores. These findings suggest that students who experienced stronger gameful engagement reported more positive overall perceptions of the escape room experience and higher perceived educational efficacy. Notably, Absence of Negative Affect was not significantly associated with educational efficacy, indicating that while minimizing negative emotions may support a positive experience, it alone may be insufficient to support perceptions of learning effectiveness.

Multiple regression analyses provided further insight into the unique contributions of individual gameful experience dimensions. Across models, Enjoyment was strongly associated with students’ evaluations of both overall perception and educational efficacy. This suggests a close relationship between affective engagement and perceived educational value. When students experience an activity as intrinsically enjoyable, they may be more motivated, attentive, and receptive, leading to more favorable evaluations of its educational value [28,34]. This result aligns with prior literature highlighting enjoyment as a core mechanism through which game-based learning influences motivation and perceived learning outcomes [28].

The strong association between enjoyment and perceived educational value is consistent with several theoretical perspectives on learning. Within Kolb’s experiential learning framework, positive affective states during an activity support the reflective observation and active experimentation stages that are central to meaningful learning [4]. When students find an activity enjoyable, they are more likely to remain engaged [34], process content more deeply, and connect new information to prior knowledge [3]. From a constructivist perspective, enjoyment may reduce cognitive resistance and support the collaborative knowledge-building processes that escape rooms are specifically designed to promote [5,6]. These variables represent closely related dimensions of learners’ subjective experience, and therefore the observed associations should be interpreted as reflecting shared experiential variance rather than independent causal effects. Given that all measures were self-reported and collected at a single time point, the findings may also be influenced by common method variance.

Dominance was also associated with higher educational efficacy perceptions, indicating that students’ sense of competence, achievement, and successful task completion played a meaningful role in shaping students’ evaluations. This finding suggests that escape rooms that clearly scaffold challenges and provide opportunities for success may be valuable in supporting perceived educational gains. Creative Thinking demonstrated a small but noteworthy contribution, suggesting that activities that stimulate problem-solving and creative reasoning may enhance perceived educational value, even if their effects are more subtle or context-dependent.

Interestingly, dominance was not significantly associated with overall DERPS perception scores, yet emerged as a significant factor of educational efficacy in the multivariable model. This pattern may reflect the conceptual distinction between the 2 outcomes. The overall perception score captures broader aspects of the escape room experience, including enjoyment, teamwork, and general attitudes toward the activity, whereas the educational efficacy subscale focuses more specifically on perceived learning value. Furthermore, regression analyses estimate the unique contribution of each experiential dimension while accounting for shared variance among dimensions. Therefore, dominance may contribute specifically to students’ perceptions of educational benefit even when its bivariate association with broader evaluations remains modest.

The association between dominance and perceived educational efficacy may be interpreted through the lens of self-determination theory [35], which identifies competence as one of the three fundamental psychological needs underlying intrinsic motivation. According to the self-determination theory, learners are more likely to perceive an educational activity as valuable when they experience a sense of effectiveness, achievement, and successful task completion. The escape room required participants to progressively solve challenges and apply knowledge under time constraints, potentially fostering feelings of competence and accomplishment. Consequently, students who reported higher dominance may also have perceived greater educational value because the activity provided opportunities to demonstrate and reinforce their capabilities in a supportive and engaging environment.

When compared with findings from other university-based educational escape room interventions, particularly in nursing education, the present results are broadly consistent with previously reported patterns of high engagement and positive gameful experience. For example, Fagundo-Rivera et al [36] reported high levels of motivation, engagement, and positive experiential outcomes among undergraduate nursing students participating in an escape room activity, with similarly elevated scores in enjoyment-related dimensions and cooperative engagement. In a subsequent mixed-methods study, the same research group further demonstrated that nursing students perceived escape room activities as highly motivating and engaging learning experiences that supported active participation and collaborative learning, although emotional challenge and task difficulty were also noted as contributing factors to the overall experience [37].

These findings are aligned with the present study, where GAMEX dimensions such as Enjoyment, Creative Thinking, and Activation were rated above the midpoint, indicating a similarly strong positive experiential profile in a medical student population. Notably, the consistency of high enjoyment and engagement across both nursing and medical education contexts suggests that these experiential dimensions may be robust features of escape room–based learning activities across health professions education, despite differences in curriculum structure, participant background, and implementation design.

In contrast, Absorption, Activation, and Absence of Negative Affect were not significantly associated with educational efficacy when considered alongside other dimensions. Regarding the Absence of Negative Affect dimension specifically, it should be noted that lower scores reflect the presence of some degree of challenge-related negative emotions rather than an entirely negative experience. As this dimension captures the extent to which participants experience low levels of frustration or anxiety during the activity, scores below the midpoint indicate that such emotions were present to some degree, which is consistent with the intentional design of escape rooms to incorporate challenge, uncertainty, and time pressure [22]. The presence of some challenge-related affect may therefore be understood as an inherent feature of the escape room format rather than evidence of an unsuccessful learning experience, and remains compatible with the strongly positive engagement and perceived educational value reported across other dimensions. Although Absorption, Activation, and Absence of Negative Affect were positively related to educational perceptions at the bivariate level, they did not uniquely explain variance in the multivariate models. This pattern suggests that these dimensions may contribute indirectly to perceived educational value by supporting enjoyment and dominance, rather than functioning as primary independent drivers of educational evaluations. The overlap among these experiential dimensions further highlights the importance of distinguishing between shared and unique components of gameful engagement.

Collectively, the findings indicate that not all dimensions of gameful experience contribute equally to students’ overall perceptions and perceived educational value. Affective enjoyment and competence-related experiences appear to be the most influential, while creative cognitive engagement plays a secondary but meaningful role. These results are consistent with broader gamification literature emphasizing that emotionally engaging, dominance-oriented learning environments can support motivation, deepen involvement, and support positive educational outcomes [35,38].

From a practical perspective, these findings offer guidance for educators seeking to design or implement educational escape rooms. Emphasizing intrinsic enjoyment, providing opportunities for dominance, and incorporating creative problem-solving elements may optimize both engagement and perceived educational value. At the same time, minimizing excessive frustration and emotional overload may help maintain a supportive learning environment without necessarily driving learning perceptions on its own. Overall, the present study contributes to the growing body of evidence supporting escape rooms as effective, learner-centered pedagogical tools in higher education [22].

The integration of medical educational escape rooms, particularly those incorporating digital elements as in our study, into medical curricula raises important considerations regarding their optimal placement as either core or extracurricular learning activities. The findings of the present study suggest that enjoyment was associated strongly with the perceived educational value, highlighting the central role of engagement in students’ learning experiences. This is especially relevant for contemporary learners, often described as Generation Z, who tend to favor interactive, technology-enhanced, and experiential learning environments [39,40]. The escape room incorporated several digital elements, including multimedia prompts, QR-based interactions, and screen-based challenges. Although their specific contribution was not evaluated separately in the present study, these features may have supported interactivity and immersion and warrant further investigation in future research [41]. From a curricular perspective, embedding escape rooms within formal teaching may support student motivation and the application of theoretical knowledge in interactive, team-based scenarios [23,42]. However, the present study did not compare different implementation formats or assess the impact of curricular integration. Escape rooms may also be used as supplementary learning experiences outside formal curricula, allowing students to engage with clinical reasoning and teamwork in a low-stakes and voluntary environment [43]. Such an approach may be beneficial for fostering creativity and minimizing negative affect, which were associated with perceived educational value in the present study [42,44]. This flexibility may be advantageous in the context of dense medical curricula. Overall, different modes of implementation (eg, curricular, extracurricular, and formative) were not directly examined in this study. Future research should compare these approaches to determine how curricular integration influences engagement, perceived educational value, and objective learning outcomes.

### Limitations and Future Research

Despite its contributions, this study has several limitations. First, outcomes were based on self-reported perceptions rather than objective measures of learning, which may introduce response bias. Future studies should incorporate knowledge tests or performance-based assessments. Second, the cross-sectional design limits causal inference. Longitudinal or experimental designs are needed to clarify the directionality and durability of observed relationships. Third, the study was conducted in a single institutional context using 1 escape room activity, which may limit generalizability. Replication across settings and disciplines is recommended. Fourth, a related limitation concerns the misalignment between the escape room’s stated learning objectives and the outcomes assessed in this study. Although the intervention was designed to support knowledge acquisition, clinical skills development, teamwork, and critical thinking, none of these domains were directly or objectively measured. The study relied on students’ self-reported perceptions of educational value, which reflect subjective evaluations rather than evidence of actual learning gains. Future studies should incorporate objective knowledge assessments or performance-based measures to more directly evaluate the educational impact of escape room interventions. In addition, potential influencing factors such as prior gaming experience, group dynamics, and individual learning styles were not examined. These may affect engagement and perceived outcomes.

A further limitation is the conceptual overlap and reliance on self-reported measures of related experiential constructs (DERPS, GAMEX, and the composite evaluation score), which were all collected at a single time point. This introduces the possibility of common method variance, which may have inflated observed associations between variables. As such, the regression analyses should be interpreted as examining relationships among closely related perceptual constructs rather than independent causal effects. Also, although the DERPS items are generic and suitable for broader educational escape room contexts, the original scale name (developed for diabetes education) may lead to potential confusion or a perceived contextual mismatch when applied in nondiabetes settings such as the present study. Finally, the study assessed immediate perceptions rather than long-term retention or behavioral change. Future research should examine whether experiential benefits translate into sustained learning outcomes.

### Conclusions

This study contributes to the growing literature on game-based learning in medical education by systematically examining both learner perceptions and dimensions of gameful experience within a student-designed educational escape room. In contrast to many previous studies that relied primarily on satisfaction-based evaluations or small-scale implementations, the present study used validated instruments and multivariate analyses to identify the experiential factors most strongly associated with perceived educational value. The findings suggest that enjoyment is strongly associated with both overall perceptions of the escape room experience and perceived educational efficacy, whereas dominance appears to be more specifically associated with perceived educational efficacy.

The findings also indicate that a digitally enriched, student-designed escape room was positively received by preclinical medical students and was associated with a favorable emotional and cognitive learning experience. By combining collaborative problem-solving, clinical simulation, and immersive gameplay, educational escape rooms may support learner engagement, motivation, and perceived educational value while complementing traditional instructional approaches. More broadly, these findings highlight the importance of considering the quality of learners’ experiences when designing gamified educational activities and provide practical guidance for the development of theory-informed, learner-centered interventions in contemporary medical curricula. These findings suggest that gameful experience dimensions are associated with students’ perceived educational value rather than objective learning outcomes.

## Abbreviations

CPR: cardiopulmonary resuscitation
DERPS: Diabetes Escape Room Perception Scale
GAMEX: Gameful Experience Scale
